# Effects of CD49d-targeted antisense-oligonucleotide on α4 integrin expression and function of acute lymphoblastic leukemia cells: Results of *in vitro* and *in vivo* studies

**DOI:** 10.1371/journal.pone.0187684

**Published:** 2017-11-08

**Authors:** Yann Duchartre, Stefanie Bachl, Hye Na Kim, Eun Ji Gang, Solah Lee, Hsiao-chuan Liu, Kirk Shung, Ruth Xu, Aaron Kruse, George Tachas, Halvard Bonig, Yong-Mi Kim

**Affiliations:** 1 Department of Pediatrics, Division of Hematology and Oncology, Children’s Hospital Los Angeles, University of Southern California Keck School of Medicine, Los Angeles, United States of America; 2 Institute for Transfusion Medicine and Immunohematology, Goethe University, and German Red Cross Blood Service Baden-Württemberg-Hessen, Frankfurt, Germany; 3 Department of Biomedical Engineering, University of Southern California, Los Angeles, United States of America; 4 Department of Pathology, University of Southern California, Los Angeles, United States of America; 5 Antisense Therapeutics Limited, Toorak, Victoria, Australia; 6 Department of Medicine, Division of Hematology, University of Washington, Seattle, WA, United States of America; University of Texas MD Anderson Cancer Center, UNITED STATES

## Abstract

We recently demonstrated the effectiveness of blocking CD49d with anti-functional antibodies or small molecule inhibitors as a rational targeted approach to the treatment of acute leukemia in combination with chemotherapy. Antisense oligonucleotide promises to be no less specific than antibodies and inhibitors, but more interesting for pharmacokinetics and pharmacodynamics. We addressed this using the published CD49d antisense drug ATL1102. *In vitro*, we incubated/nucleofected the ALL cell line Kasumi-2 with ATL1102. *In vivo*, immunodeficient hosts were engrafted with primary ALL cells and treated with ATL1102. Changes in expression of CD49d mRNA and CD49d protein, and of cooperating gene products, including ß1 integrin and CXCR4, as well as survival in the mouse experiments were quantified. We observed dose-dependent down-regulation of CD49d mRNA and protein levels and its partner integrin ß1 cell surface protein level and, up-regulation of CXCR4 surface expression. The suppression was more pronounced after nucleofection than after incubation, where down-regulation was significant only at the higher doses. *In vivo* effects of ATL1102 were not sufficient to translate into “clinical” benefit in the leukemia model. In summary, antisense oligonucleotides are successful tools for specifically modulating gene expression but sufficient delivery to down-regulate CD49d *in vivo* may be difficult to achieve.

## Introduction

Childhood Acute Lymphoblastic Leukemia (ALL) is characterized by an overproduction of lymphoblasts which accumulate mainly in bone marrow (BM), peripheral blood and spleen. Despite efficient treatments for most patients (chemotherapy in particular inducing complete remission in ~80% of pediatric patients), drug resistance and subsequent relapse represents an important issue. This, as well as serious off-target toxicity, occasionally dose-limiting, fuel the quest for novel treatment strategies. Relapse is caused by leukemic cells surviving chemotherapy treatment, which is mediated at least in part via chemoprotective interactions between leukemia (stem) cells and BM stroma[1]. The α4 integrin (CD49d/ITGA4) (subunit of the adhesion molecule very late antigen 4, (VLA-4) with its partner the integrin β1 (CD29)) directly interacts with several components of the BM microenvironment, including VCAM-1, fibronectin and osteopontin, and regulates many cellular functions including signal transduction, proliferation and adhesion[2, 3]. The α4 integrin has been identified as a major player in cell adhesion-mediated drug resistance (CAM-DR) in AML[4]. Blockade of CD49d using specific antibody (Natalizumab) sensitized resistant ALL to chemotherapy, underscoring the potential of α4 integrin targeting therapies as an avenue to abolish the chemoprotective effect of the microenvironment on ALL cells[5]. A potential disadvantage of anti-functional antibodies is their much longer than needed bioavailability. While month-long blockade of CD49d is useful for treatment of chronic inflammatory diseases, for leukemia treatment short-acting blockade only for the duration of the cytotoxic effects of the chemotherapy would be sufficient and possibly preferable [6]. Seeking to identify compounds to address this, we tested a novel second-generation antisense oligonucleotide to CD49d RNA named ATL1102. This antisense oligonucleotide was developed for the treatment of multiple sclerosis (MM) and suggested efficacy in patients with relapsing-remitting multiple sclerosis (RRMS) in Phase 2 trials, in spite of a limited reduction of VLA-4 expression[7]. Although there was no evidence that relevant amounts of ATL1102 managed to enter the target cells, in vivo mice studies with ATL1102 indicated distribution of the antisense to lymphoid organs including bone marrow, spleen and lymph nodes [7] like all other antisense drugs of this class, suggesting favorable pharmacokinetics for the present studies. ATL1102 is specific to human CD49d RNA and is not complementary to mouse CD49d RNA. Here we evaluated the effects of ATL1102 on chemoresistant human ALL cells *in vitro* and *in vivo* in mice.

## Methods

### *In vitro* targeting of CD49d expression in Kasumi-2 cell line

3x10^6^ Kasumi-2 cells were nucleofected with a control antisense (30μM) or CD49d antisense (ATL1102: 1μM, 3μM, 10μM and 30μM) using Amaxa Nucleofector Kit V (Lonza, according to manufacturer’s protocol). Briefly, two days after last seeding, Kasumi-2 cells were counted and 1 million cells per condition were centrifuged at 100g for 10 minutes at room temperature. After complete removal of the supernatant, the cells’ pellet was resuspended in 100μl room temperature mixed Nucleofector® solutions. After addition of the antisense compound, cells were nucleofected using Nucleofector® device. Immediately, cells were mixed with 500μl of culture medium and plated in a 12-well plate. CD49d expression was assessed by flow cytometry every 24h after treatment for 72h using specific human CD49d antibody (clone 9F10, eBiosciences). Other antibodies (Biolegend, PE or APC- conjugated) used for flow cytometry included anti-human CD19 (HIB19), anti-human CD29 (TS2/16), anti-human CD49e (NKI-SAM-1), anti-human/mouse CD49f (GoH3) and anti-human CXCR4 (12G5).

### Effects of ATL1102 on apoptosis in Kasumi-2 cell line

3x10^6^ of Kasumi-2 cells were nucleofected as previously described. Apoptosis assay was performed using double staining AnnexinV (PE) and DAPI and assessed by flow cytometry every 24h after treatment for 72h.

### Quantitative real-time PCR

RNA extraction (RNeasy Plus Mini Kit, Qiagen) and RNA retro-transcription (SuperScript III First-Strand Synthesis System, Invitrogen) were performed on Kasumi-2 frozen pellets at 24h, 48h and 72h post treatment. Quantitative real-time PCR was performed by mixing cDNA samples with the SYBR GreenER (Invitrogen) and analyzed by ABI7900HT real-time PCR system (Applied Biosystems). Results are presented as a ratio on control antisense-treated cells. *In vivo*, total bone marrow cells were frozen down. hCD49d expression was determined by specific primers for human integrin alpha 4 and integrin beta 7.

*hIntegrin alpha4_Fw*                    5’-CCCCAGGATCATCTTACTGGA-3’
*hIntegrin alpha4_Rv*                    5’-TATGCTGGCTCCGAAAATGAC-3’
*hIntegrin beta7_Fw*                     5’- ACAGTTGCATCAGTCCCGAG-3’
*hIntegrin beta7_Rv*                     5’- AGCTGTACTGCAGTTGGTGG-3’
hGAPDH_Fw                              5’-GTTGCCATCAATGACCCCTTCATTG-3’
hGAPDH_Rv                              5’-GCTTCACCACCTTCTTGATGTCATC-3’

### Western blotting

One million cells were washed twice in cold PBS and lysed on ice in M-PER buffer (ThermoFisher Scientific, Rockford, IL) supplemented with a 1% protease inhibitor cocktail (Pierce, Rockford, IL). Proteins were separated by SDS-PAGE and electro-transferred to PVDF membrane (Invitrogen, Carlsbad, CA). We used anti-Integrin α4 (Cell Signaling Technology, Danvers, MA) and β-Actin (C-4) (Santa Cruz Biotechnology, Santa Cruz, CA) as primary antibodies to reveal those proteins. Alkaline phosphatase conjugated anti-rabbit secondary antibody solution (ThermoFisher Scientific, Rockford, IL) was used to detect the primary antibodies.

### *In vivo* ATL1102 treatments

Under protocols approved by CHLA’s IACUC, 5x10^6^ of ALL patient sample cells (LAX7R) were injected via the tail vein in sublethally irradiated NOD.Cg-Prkdcscid IL2rgtm1Wjl/SzJ (NSG) female mice (n = 28) of 5–7 weeks of age (single sub-lethal dose of 250 cGy whole body irradiation. 7 days after xenografting, the mice were treated with different regimens: control (PBS) (n = 3); combination of Vincristine, Dexamethasone and L-Asparaginase (VDL) 5 times/week intraperitoneally for 5 weeks (n = 4); ATL1102 (150mg/kg once per week by intravenous (IV) injection (n = 4) or 3x50mg/kg/week by subcutaneous (SC) injection (n = 3)) or the combinations VDL + ATL1102 (IV; n = 7; or SC; n = 7). Weight change was monitored for the total duration of the experimentation (77 days for survival study and 3 weeks for the target effect experimentation) and leukemia engraftment and progression were determined by presence of human cells (hCD45) in the peripheral blood which was assessed by FACS and bioimaging[8, 9].

#### Mice

NOD.Cg-Prkdcscid IL2rgtm1Wjl/SzJ (NSG) mice as primary pre-B ALL recipients were previously described[5]. Mice were bred and housed at the comparative medicine facility of CHLA under SPF conditions in microisolator cages, with autoclaved chow and water ad libitum. Only female mice, aged between 5–7 weeks, were used for *in vivo* leukemia studies.

#### Ethics and approvals

Primary patient material were de-identified and re-coded and used for xenografting and approved by the Children’s Hospital Los Angeles Institutional Review Board (Approved protocol # CCI-08-00102). For animal studies, animals were monitored and observed daily or every other day. Weight (loss of 15% of body weight) and animal behavior were used as humane endpoints. Out of 28 animals all mice were found dead before this endpoint was observed. Animal studies were approved by CHLA’s IACUC.

#### Statistics

Descriptive statistics and Student’s t-tests, corrected for multiple testing using Bonferroni correction, were calculated in excel (Microsoft, Redmond, WA). Data are given as mean±SEM unless otherwise indicated. Survival in the different cohorts was compared using the log-rank test. A p<0.05 was considered statistically significant. Unless otherwise indicated, n≥3 independent experiments were performed for all outcomes. (T-test: All conditions vs CTRL, ns p > 0.05, * p ≤ 0.05, ** p ≤ 0.01, *** p ≤ 0.001, **** p ≤ 0.0001).

## Results

### CD49d surface expression decreases in ATL1102-nucleofected Kasumi-2 ALL cells

**Fig 1:**
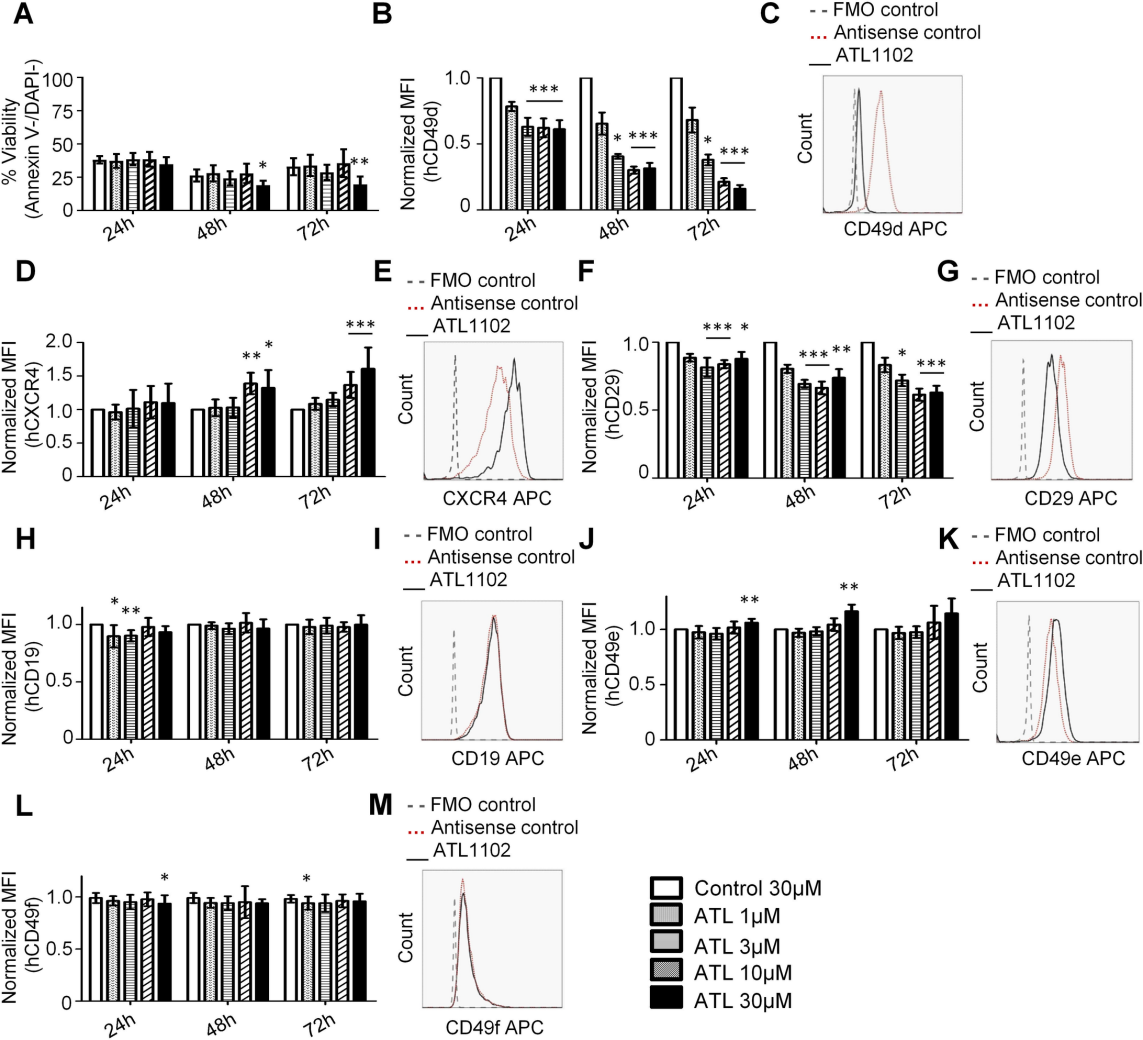
Nucleofection or incubation of ALL cell line *Kasumi-2* with the antisense ATL1102. **ALL cells were treated with various concentrations of ATL1102 (1** μ**M, 3** μ**M, 10** μ**M and 30** μ**M) or 30**μ**M control as indicated by grey bars. A.** Viability of Kasumi-2 cells at 24h, 48h and 72h post nucleofection. **B-M.** Expression of surface proteins determined by flow cytometry 24h, 48h and 72h postnucleofection. ***B-C***. CD49d. ***D-E*.** CXCR4. ***F-G***. CD29. ***H-I***. CD19. ***J-K*.** CD49e. ***L-M***. CD49f. Histograms show the surface marker expressed under nucleofection with the highest dose of ATL1102 (30 μM) compared to antisense control. (Analysis performed by ANOVA one way; *p<0.05, **p<0.01, ***p<0.001).

To study ATL1102 effects on ALL cells, the ALL cell line Kasumi-2 was treated with the CD49d RNA antisense oligonucleotide for 3 periods of time. In order to optimize the effects of ATL1102, we used nucleofection to deliver the antisense directly into the cells and potentiate its effects. Previous experiments had established doses in the low μM range as effective (data not shown); we therefore used concentrations of 1μM, 3μM, 10μM and 30μM. Nucleofection caused significant cell death which was, however, attributable to the manipulation and not specific to ATL1102 (Fig 1A). In the surviving cells, ATL1102 treatment down-regulated CD49d protein expression; kinetics was rapid, with statistically significant down-regulation observed as early as 24 h after transfection (Fig 1B and 1C). The effect was sustained for at least 72h, indicating high ATL1102 stability/persistence in the cell. Interestingly, surface expression of CXCR4 was markedly and dose-dependently up-regulated in ATL1102 treated cells, a mechanism possibly attributable to compensatory attempts by CD49d-depleted cells (Fig 1D and 1E). We concurrently observed downregulation of CD29 (integrin beta1), the predominant ß-partner of CD49d on hematopoietic cells (Fig 1F and 1G). In addition, downregulation of the less dominant partner of CD49d, integrin beta7, was downregulated as well as assessed by RT-PCR (S1 Fig). As expected of a compound of this class, ATL1102 showed high specificity of action for the integrin alpha4, in that it did not affect the expression of other integrins like integrin alpha5/CD49e (Fig 1J and 1K) and integrin alpha6/CD49f (Fig 1L and 1M). We also checked if ATL1102 could modify CD19 expression which could reduce the efficacy of a CD19 targeting therapy. No such change was observed (Fig 1H and 1I).

### CD49d surface expression decreases in ATL1102-incubated Kasumi-2 ALL cells

**Fig 2:**
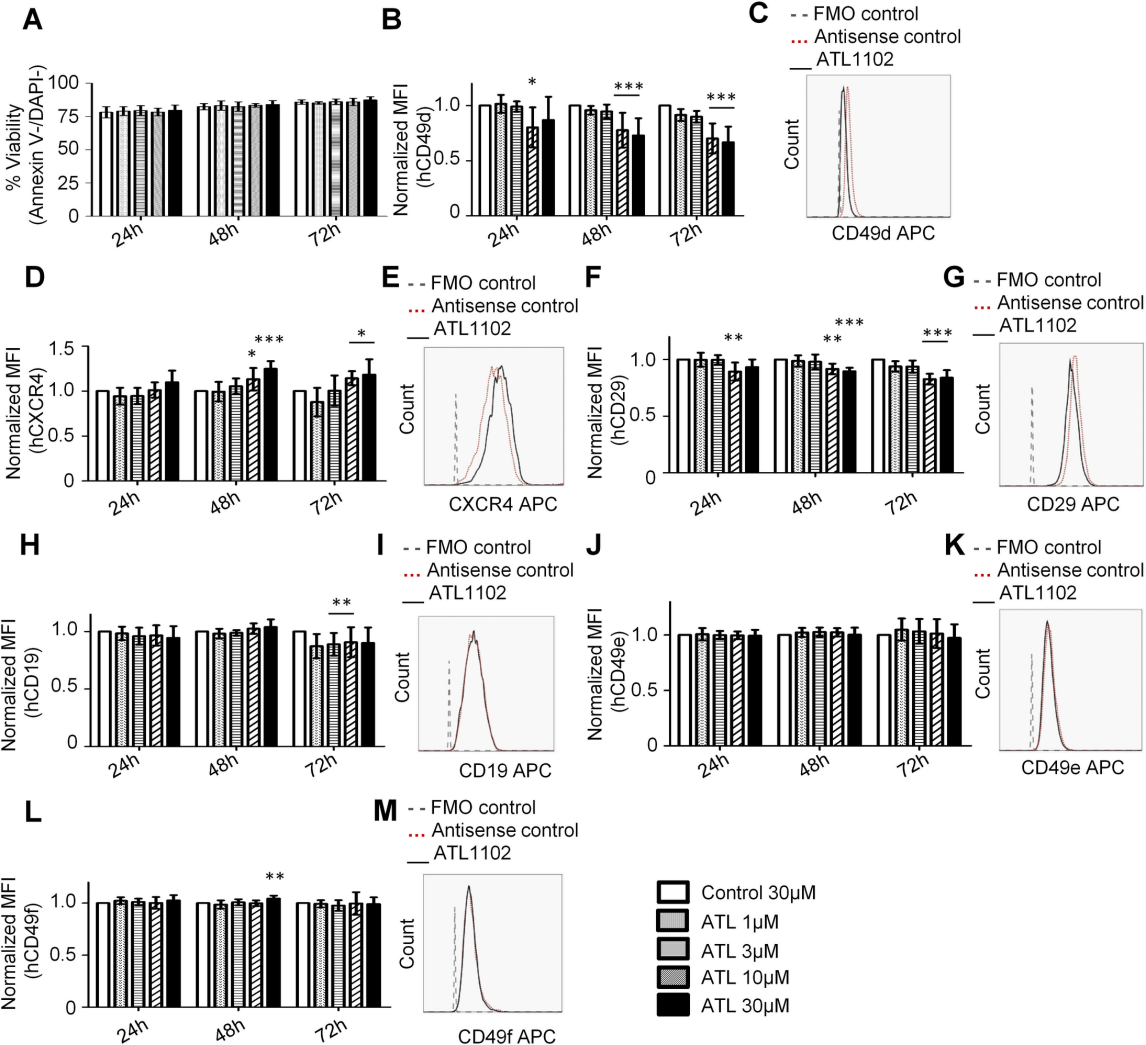
Incubation of ALL cell line *Kasumi-2* with the antisense ATL1102. **ALL cells were incubated with various concentrations of ATL1102 (1** μ**M, 3** μ**M, 10** μ**M and 30** μ**M) or 30**μ**M control as indicated by grey bars. A.** Viability of Kasumi-2 cells at 24h, 48h and 72h post incubation. **-*B-M*.** Expression of surface proteins determined by flow cytometry 24h, 48h and 72h post incubation. ***B-C***. CD49d. ***D-E*.** CXCR4. ***F-G***. CD29. Expression of surface proteins determined by flow cytometry 24h, 48h and 72h post incubation. ***H-I***. CD19. ***J-K*.** CD49e. ***L-M***. CD49f. Histograms show the surface marker expressed under incubation with the highest dose of ATL1102 (30 μM) compared to antisense control. (ANOVA one way *p<0.05, **p<0.01, ***p<0.001).

Since as a pharmacological substance, ATL1102 is injected systemically and must spontaneously pass through the cell membrane in order to elicit therapeutic responses, we sought to model the efficacy of this by simply incubating Kasumi-2 cells in ATL1102 at concentrations which can reasonably be achieved *in vivo*. Unlike nucleofection, co-incubation was not associated with toxicity at 24, 48 and 72 hours of treatment (Fig 2A). These incubation experiments qualitatively fully corroborated the findings of the nucleofection experiments, although all effects were quantitatively attenuated by comparison. Thus incubation of Kasumi-2 with ATL1102 led to dose-dependent decrease of CD49d surface expression compared to antisense control-treated cells (Fig 2B and 2C). Also as in the nucleofection experiments, albeit quantitatively less pronounced, CXCR4 surface expression on ATL1102 incubated Kasumi-2 cells trended towards higher levels in an ATL1102 dose-dependent manner (Fig 2D and 2E). CD29 expression also trended towards lower levels with increasing concentrations of ATL1102 (Fig 2F and 2G). As in the nucleofection experiments ATL1102 did not modify expression of the other integrins CD49e (Fig 2J and 2K) and CD49f (Fig 2L and 2M), nor expression of CD19 (Fig 2H and 2I). To determine if CXCR4 is downregulated with a different method of CD49d interference, we treated Kasumi-2 cells with Natalizumab for 48h and 72 hours, and we did not observe a change in CXCR4 MFI (S2 Fig). Natalizumab binding to Kasumi-2 was confirmed by flow cytometry (S3 Fig).

### CD49d protein expression decreases in both ATL1102-incubated and nucleofected Kasumi-2 ALL cells

**Fig 3:**
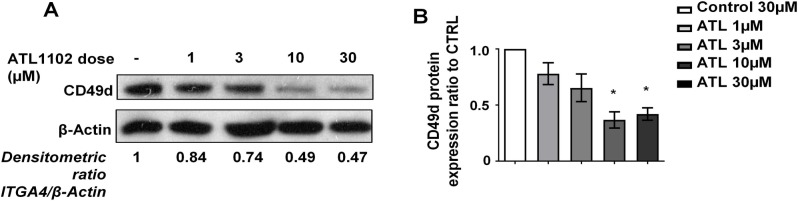
CD49d expression determined by Western Blot 72h post nucleofection. Kasumi-2 cells were treated with antisense control (**-**), 1μM (**1**), 3 μM (**3**), 10 μM (**10**) or 30 μM (**30**) of ATL1002 and optical density was determined (normalized to control). **A.** One representative Western Blot of at least three independent experiments is shown. **B**. Mean optical density (n = 3, normalized to control) (ANOVA one way *p<0.05). Western Blot lysates from incubated Kasumi 2 cells qualitatively confirmed the observations of flow cytometric analyses, although because of high inter-assay variability did not achieve statistical significance.

In order to dissect whether the above-described effect of ATL1102 on CD49d expression was due to total protein expression vs. surface presentation, we analyzed by Western Blot total cell extracts from ATL1102 nucleofected (Fig 3A and 3B)- or incubated-cells (data not shown). Both nucleofection and incubation reduced CD49d total expression in a dose-dependent and ATL1102 presentation-dependent manner, i.e. more strongly in nucleofected than in incubated cells.

### The CD49d downregulation is due to CD49d RNA targeting

**Fig 4:**
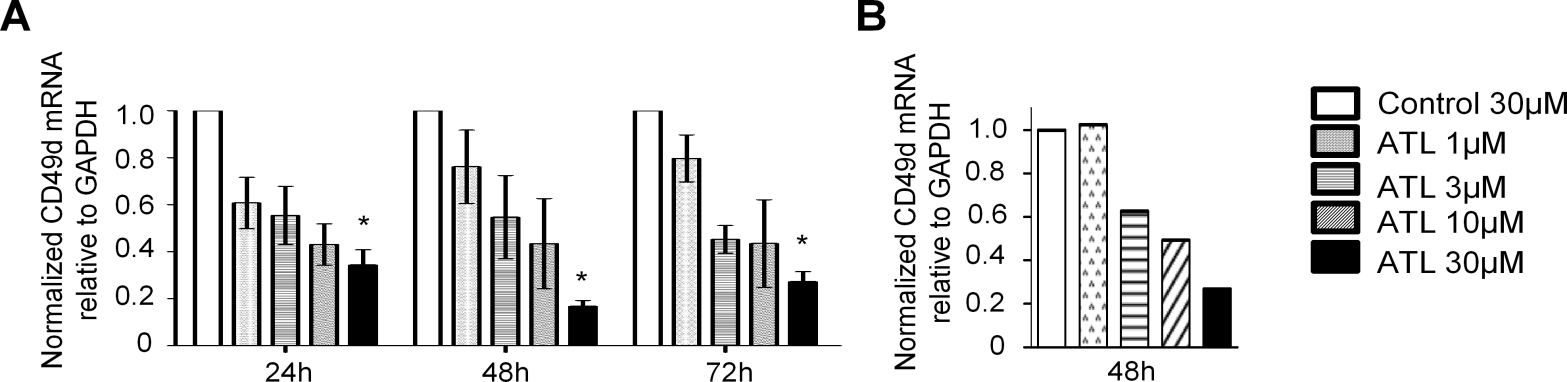
Quantitative RT-PCR post nucleofection / incubation. **A.** Expression of CD49d in Kasumi-2 cells nucleofected with ATL1102 after 24h, 48h and 72h. **B.** Expression of CD49d in Kasumi-2 cells incubated with ATL1102 after 48h (n = 1). For technical reasons, RT-PCR for incubated cells was only performed once but observations agreed with the concurrently generated flow cytometry data as well as with the data generated by electroporating with ATL1102.

After nucleofection of Kasumi-2 cells with 5 different concentrations of the ATL1102 compound, we checked CD49d mRNA expression by quantitative real-time PCR (Fig 4). We determined expression of CD49d mRNA relative to the expression of GAPDH at 24h, 48h and 72h. Nucleofection induced a dose-dependent downregulation of CD49d expression starting from 24h (Fig 4A). This effect starts vanishing at 72h, possibly due to ATL1102 degradation.

### CD49d expression is not affected by *in vivo* ATL1102 treatment in a mouse model of B-ALL

**Fig 5:**
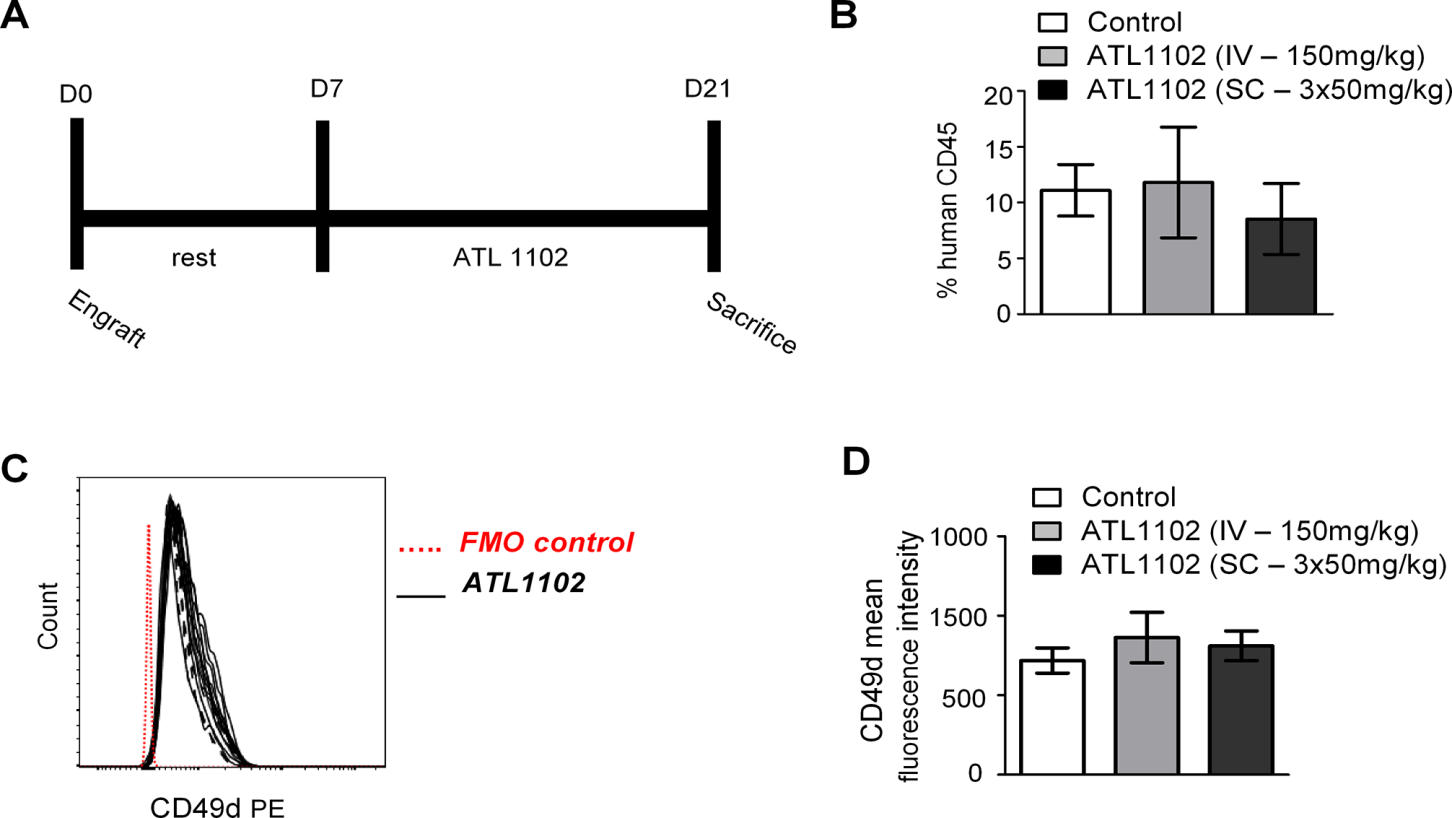
In vivo ATL1102 *target effect* study in mouse xenograft model of B-ALL. NOD/SCID mice were engrafted with primary B-ALL cells LAX7R, then treated with ATL1002 for two weeks (group 1: s.c., group 2: i.v. n = 5 each group) **(A)**. The mice were sacrificed 3 days after the last s.c. or i.v. injection. After sacrifice, percentage of hCD45 (**B**) and CD49d mean fluorescence intensity (**C, D**) was determined by flow cytometry in peripheral blood.

Once the efficacy of ATL1102 to down-regulate CD49d was determined in vitro, we sought to test its efficacy *in vivo*. We xenografted immunodeficient (NSG) mice with human primary B-ALL cells from our laboratory (LAX7R). One week after leukemia cell transfer, we treated mice with 2 regimens of ATL1102 (150mg/kg via intravenous way once a week or 50mg/kg subcutaneously three times a week) or with an identically dosed control antisense (Fig 5A). After two weeks of treatment, we analyzed the frequency of LAX7R cells in the recipients’ blood by enumerating hCD45^+^ vs. mCD45^+^ cells to determine if ATL1102 can effectively reduce the CD49d expression. While subcutaneous injection of ATL1102 seemed to reduce the percentage of LAX7R cells (Fig 5B), there was no downregulation of CD49d *in vivo* (Fig 5C and 5D).

### *In vivo* ATL1102 treatment does not improve survival in a mouse model of B-ALL

**Fig 6:**
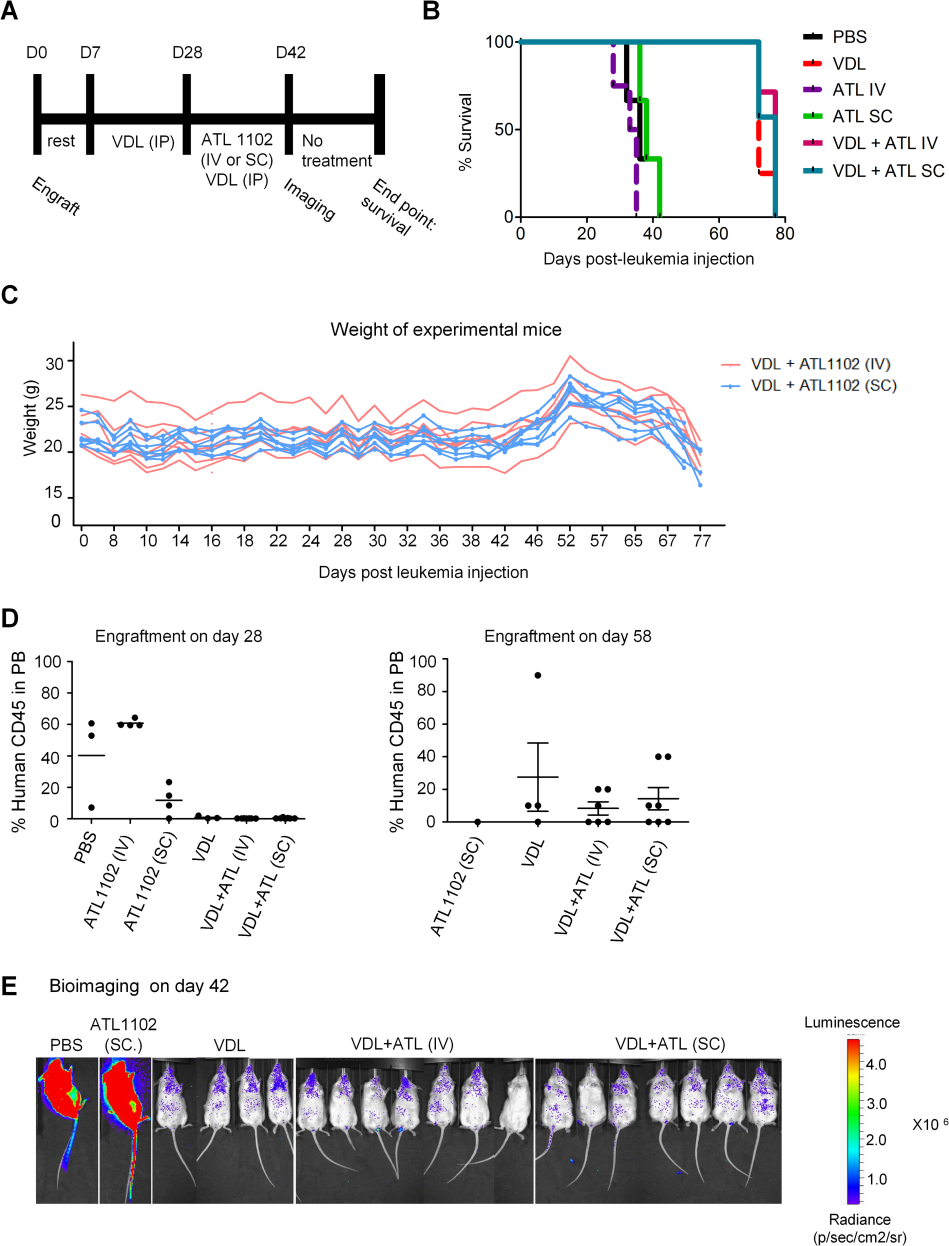
In vivo ATL1102 *survival* study in mouse xenograft model of B-ALL. Mice were engrafted with LAX7R similarly as described previously[5] and treated for four weeks with saline (control group), VDL (Vincristine, Dexamethasone, L-Asparaginase) (IP), a combination of VDL (IP)+ ATL1102 (IV or SC) or ATL1102 (IV or SC) only (**A**). Kaplan-Meier survival curve of NOD.Cg-Prkdc^scid^ IL2rg^tm1Wjl^/SzJ (NSG) recipients in days post engraftment (**B**).Weight evolution during VDL or/and ATL1102 treatment (**C**). Engraftment was determined by human CD45^+^ cells in mouse peripheral blood (PB) using flow cytometry on day 28 post-leukemia injection (left panel and day 58 post-leukemia injection (right panel) (**D**). Bioimaging was performed on day 42 post-leukemia injection (**E**).

We moreover studied the effects of ATL1102 on survival in the same leukemic mouse model in which we had previously demonstrated efficacy of the anti-functional anti-CD49d antibody Natalizumab, i.e. in combination or not with a chemotherapy consisting of Vincristin, Dexamethasone and L-Asparaginase (VDL). One week after xenograft, some mice were treated with VDL intraperitoneally for 3 weeks (Fig 6A). Four weeks after xenograft, we started the ATL1102 treatment which resulted in six different groups: PBS, VDL, ATL1102 (IV, 150mg/kg), ATL1102 (SC, 3x50mg/kg), VDL + ATL1102 (IV, 150mg/kg) and VDL + ATL1102 (SC, 3x50mg/kg). After two weeks of combinatorial treatment VDL + ATL1102 or ATL1102 alone, mice were followed for survival. The mice that were not treated with the VDL chemotherapy died quickly D32±3 ATL1102 i.v., Day 39±4 ATL1102 s.c., Day 40 ±3 PBS. VDL provided some palliative effect, but the mean survival in the three VDL groups, VDL alone, VDL+ATL i.v. and VDL+ATL s.c., all also died approximately at the same time (day 77) demonstrating that ATL1102 at the doses and routes used does not improve survival (Fig 6B). Lack of weight loss during the treatment with ATL indicates that ATL1102 was well tolerated by the recipient mice (Fig 6C). The observed weight decline after 52 days post-leukemia injection is attributable to the leukemia progression (Fig 6C). Engraftment was assessed by flow cytometric detection of human CD45^+^ MNCs in the peripheral blood of mice at day 28 (Fig 6C, left panel) and day 58 post-leukemia injection (Fig 6C, right panel). All groups showed at some point human CD45^+^ cells in the peripheral blood. Bioimaging at day 42 post-leukemia injection demonstrates also leukemia progression earliest detectable in PBS treated control mice and ATL1102 only treated mice (Fig 6E). Taken together these data point to engraftment of leukemia in this model.

## Discussion

We and others have demonstrated the potential of CD49d-targeting treatments as supplementation for cytoreductive chemotherapy in acute leukemia. The proposed mechanism of action is disruption of adhesive interactions of leukemia cells with BM stroma, stroma providing survival cues, adhesive interactions signaling negative cell cycle activity, and de-adhesion also resulting in physically better exposure to chemotherapy drugs, in aggregate resulting in more effective leukemia cell killing by chemotherapy drugs[3, 5, 10, 11]. While anti-functional antibodies for CD49d targeting are available, certain disadvantages are associated with these. Thus CD49d blockade with Natalizumab predisposes to re-activation of John Cunningham virus, resulting in potentially lethal encephalopathy (PML) [12]. This is an infrequent complication Natalizumab-treated patients with relapsing-remitting Multiple Sclerosis, but more frequent when on immunosuppressive therapy, so it could conceivably be more frequent in leukemia patients due to the therapy-inherent immunosuppression, and treatment of leukemia patient is further complicated by the long half-life of Natalizumab. The month-long bioavailability of the antibody is clinically useful for chronic diseases like Multiple Sclerosis, but for leukemia treatment presence of the inhibitor only during the chemotherapy is likely sufficient and, because of the above-said risks of PML, probably preferable. Antisense therapies promise to fulfill this criterion better, as much as they can be made highly specific with minimal or no off-target effects, provided the compound enters the target cells in pharmacologically relevant quantities. The purpose of our studies was to test specificity and efficacy of a CD49d antisense drug as a potential anti-leukemic drug, using *in vitro* and *in vivo* experiments.

As we are showing, the compound down-regulates CD49d, at the same time reducing CD29 ß1 integrin surface expression. Since solitary beta-integrin chains are not inserted in the membrane and alternative α partners for ß1 integrin are not altered in their expression, down-regulated ß1 after ATL1102 treatment is presumably the result of the lack of the predominant partner for ß1 [13, 14] [15]. Similar results were described for mice lacking CD49d [16]. Of great interest is the up-regulation of CXCR4 in ATL1102 treated leukemia cells. Several lines of evidence suggest that CD49d and CXCR4 both mediate similar cell fates, namely CAM. Certainly for normal hematopoietic cells both serve as retention mediators and their blockade, alone or in combination, leads to egress, or mobilization, of mature and immature hematopoietic species [17, 18] [19] [20, 21]. CXCR4 being a superbly targetable molecule, the potential for combinatorial targeting of CD49d and CXCR4 with antagonists for the (neoadjuvant) treatment of acute leukemia should be considered in light of our novel findings. All available data indicate a high degree of specificity of ATL1102 as well as considerable potency if transfected directly into the cells *in vitro*. When, however, ATL1102 must enter the cell spontaneously *in vitro*, the effect on CD49d is qualitatively the same, but quantitatively significantly blunted. The ATL1102-induced CD49d downregulation may be enhanced by improvement of the antisense delivery into the cells in vitro. Combining oligonucleotide with gold nanoparticules, antibody or lipid particles conjugated with polyethylene glycol (PEGylation) are examples of the successful delivery methods in vivo[22]. ATL1102-induced CD49d downregulation may be enhanced in LAX7R cells in vivo using a peptide RGD-oligonucleotide conjugate to bind to the cell surface integrins as described[22]. These observations are consistent with antisense drugs of this class *in vitro* whereas uptake *in vivo* can occur in certain cell types like hepatocytes leading to down regulation, pharmacological outcomes and clinical benefits. The *in vivo* application of ATL1102, did not however lead to down-regulation of CD49d in LAX7R cells even at high doses of ATL1102 in mouse xenograft studies. Consequently, *in vivo* application of ATL1102 in mice bearing human leukemias did not alter leukemia progression and death.

In a clinical trial in Multiple Sclerosis patients, efficacy of ATL1102 had been suggested based on significant reduction in inflammatory brain lesions associated with annualized relapse reductions and had provided the rationale for selection of the compound for our studies [7]. Do those data thus run counter to our observations? Not necessarily. In that study as in ours, there was reduction in lymphocytes attributed to VLA-4 mechanisms of survival and proliferation, but down-regulation of CD49d on lymphocytes was noted only in a small population of spared T and B-cells [7]. ATL1102 treatment was as active as Natalizumab in reducing brain lesions in similar 12 week studies but did not show other hallmarks of CD49d cell surface blockade, such as leukocytosis attributed to interference of transmigration[23]. In the absence of further mechanism of action studies, one can only speculate about explanations for the apparent clinical benefit observed in the MS patients. The chemical nature of the compound, and other naked nucleic acids, suggests that they may activate the innate immune system leading to increase neutrophils in a non-specific fashion, with the induced cytokines modulating disease activity in the autoimmune setting [24]. The opposite, however, occurred in the ATL1102 MS study, and the role of the innate immune system in RR-MS is unknown [7].

Our studies thus demonstrate the suitability of antisense drugs for highly specific mRNA transcript targeting, but at the same time identify obstacles to their clinical use at this time, namely insufficient entry into target cells to elicit potent on-target effects after systemic delivery. Clearly the results are encouraging with regards to the potential of antisense drugs but identify the need for considerable improvements of *in vivo* delivery for application in B-ALL mobilization from the bone marrow.

## Conclusion

In this study we demonstrated that the CD49d antisense ATL1102 successfully downregulates its target on human pre-B ALL cells *in vitro* but not *in vivo* in mouse xenografts. Further investigations still need to be done in order to assess the *in vivo* uptake of ATL1102 in other leukemias, but suggest a need to improve cellular delivery to reach the full potential of ATL1102 as a new therapeutic option for ALL patients.

## Supporting information

S1 Fig Expression of ITGB7 by quantitative real time PCR. Kasumi-2 cells were nucleofected with antisense ATL1102 (ATL) (1 μM, 3 μM, 10 μM or 30 μM) or antisense control (30 μM), Leukemia cells were harvested for RNA extraction. ITGB7 expression was determined by real time PCR 24h, 48h and 72h post-nucleofection. Normalized ITGB7 mRNA relative to GAPDH is shown. T-test: All conditions vs antisense control, * p ≤ 0.05, ** p ≤ 0.01, *** p ≤ 0.001.
(TIF)

S2 Fig CXCR4 expression in Kasumi-2 cells treated with Natalizumab. Cells were incubated with Natalizumab (20μg/mL) or its isotype IgG4 (20μg/mL) for 48h and 72h. **A.** Median fluorescence intensity (MFI) of CXCR4 expression normalized to Media control. **B.** Representative histogram of CXCR4 expression under different conditions compared to Media control.
(TIF)

S3 Fig Natalizumab binds to the surface of Kasumi-2 cells. Cells were incubated with 20μg/ml of Natalizumab for 30 minutes, and then washed once with PBS. CD49d was measured using Alexa Fluor 488 (Invitrogen)-labeled Natalizumab by flow cytometry. **A**. Representative dot plots and **B.** histogram of CD49d expression. FSC: Forward scatter. SSC: Side scatter. PI: Propidium iodide. **C.** Natalizumab binding in Natalizumab treated or isotype control (IgG4) treated groups. ****p<0.0001 by Two-Way ANOVA.
(TIF)
