# Supplementary figures and images for: Effects of CD49d-targeted antisense-oligonucleotide on α4 integrin expression and function of acute lymphoblastic leukemia cells: Results of in vitro and in vivo studies
Source: PLoS One. 2017 Nov 8;12(11):e0187684. doi: 10.1371/journal.pone.0187684 (PMC5678723; doi:10.1371/journal.pone.0187684)

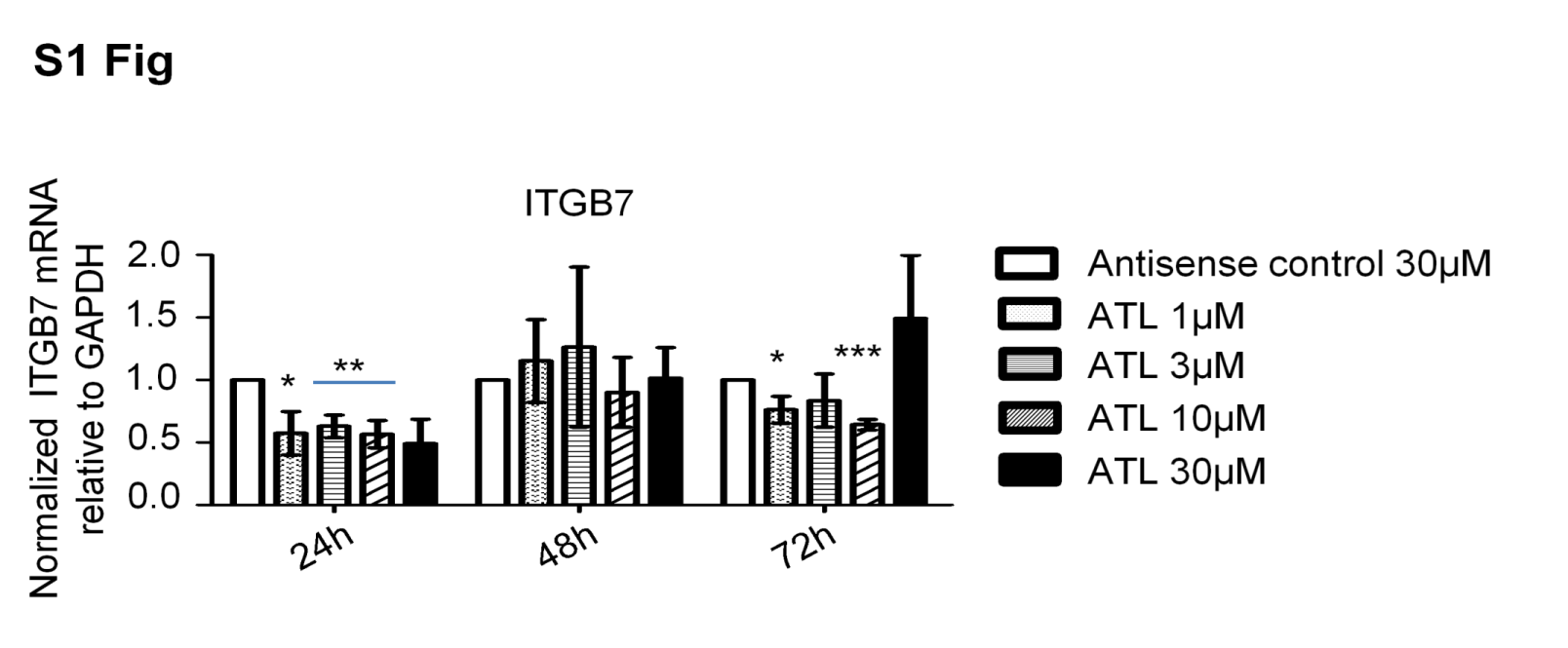

Supplement: S1 Fig — Kasumi-2 cells were nucleofected with antisense ATL1102 (ATL) (1 μM, 3 μM, 10 μM or 30 μM) or antisense control (30 μM), Leukemia cells were harvested for RNA extraction. ITGB7 expression was determined by real time PCR 24h, 48h and 72h post-nucleofection. Normalized ITGB7 mRNA relative to GAPDH is shown. T-test: All conditions vs antisense control, * p ≤ 0.05, ** p ≤ 0.01, *** p ≤ 0.001. (TIF) [file pone.0187684.s001.tif]

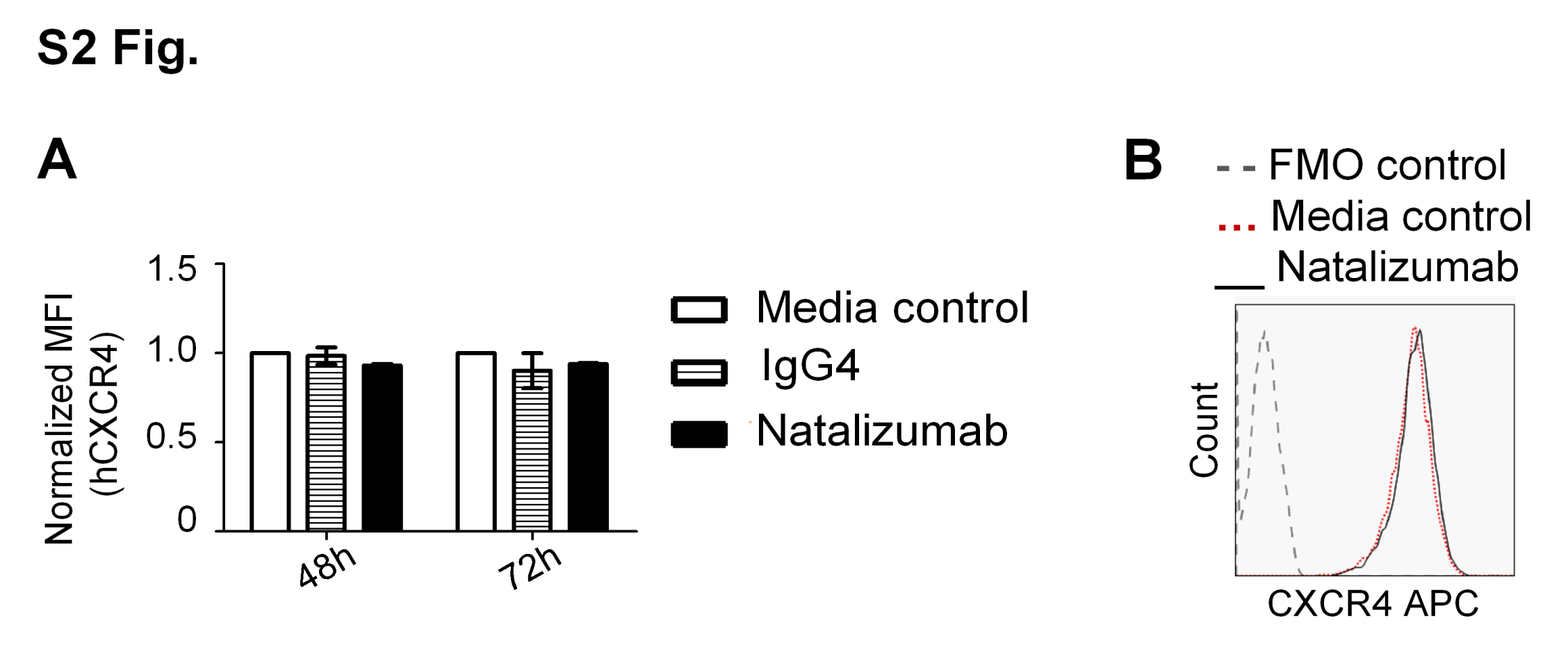

Supplement: S2 Fig — Cells were incubated with Natalizumab (20μg/mL) or its isotype IgG4 (20μg/mL) for 48h and 72h. A. Median fluorescence intensity (MFI) of CXCR4 expression normalized to Media control. B. Representative histogram of CXCR4 expression under different conditions compared to Media control. (TIF) [file pone.0187684.s002.tif]

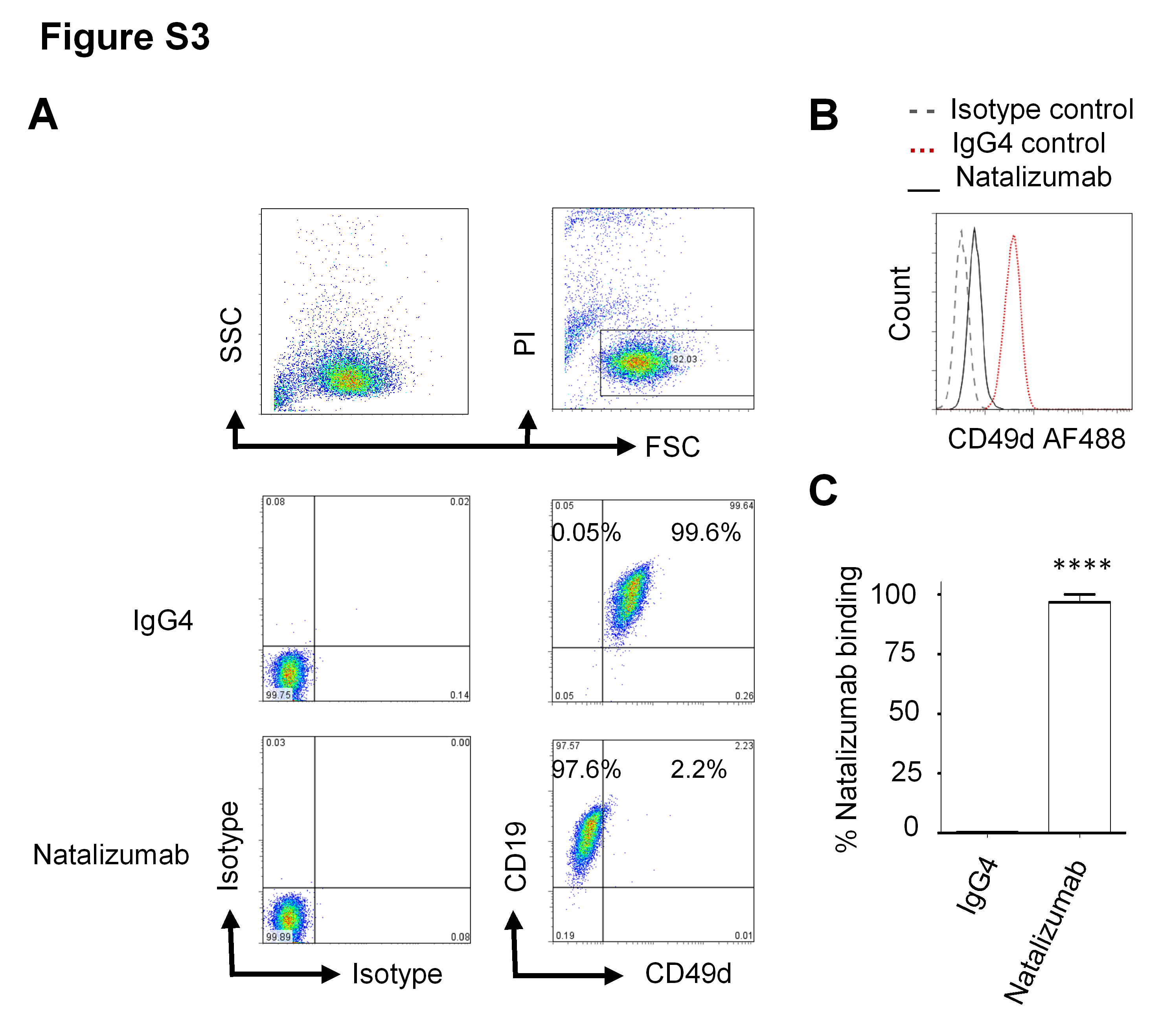

Supplement: S3 Fig — Cells were incubated with 20μg/ml of Natalizumab for 30 minutes, and then washed once with PBS. CD49d was measured using Alexa Fluor 488 (Invitrogen)-labeled Natalizumab by flow cytometry. A. Representative dot plots and B. histogram of CD49d expression. FSC: Forward scatter. SSC: Side scatter. PI: Propidium iodide. C. Natalizumab binding in Natalizumab treated or isotype control (IgG4) treated groups. ****p<0.0001 by Two-Way ANOVA. (TIF) [file pone.0187684.s003.tif]
